# Liver biomarkers, genetic and lifestyle risk factors in relation to risk of cardiovascular disease in Chinese

**DOI:** 10.3389/fcvm.2022.938902

**Published:** 2022-08-11

**Authors:** Xinyu Wang, Si Cheng, Jun Lv, Canqing Yu, Yu Guo, Pei Pei, Ling Yang, Iona Y. Millwood, Robin Walters, Yiping Chen, Huaidong Du, Haiping Duan, Simon Gilbert, Daniel Avery, Junshi Chen, Yuanjie Pang, Zhengming Chen, Liming Li

**Affiliations:** ^1^Department of Epidemiology and Biostatistics, School of Public Health, Peking University, Beijing, China; ^2^Peking University Center for Public Health and Epidemic Preparedness & Response, Beijing, China; ^3^Key Laboratory of Molecular Cardiovascular Sciences, Ministry of Education, Peking University, Beijing, China; ^4^Chinese Academy of Medical Sciences, Beijing, China; ^5^Clinical Trial Service Unit and Epidemiological Studies Unit (CTSU), Nuffield Department of Population Health, University of Oxford, Oxford, United Kingdom; ^6^Medical Research Council Population Health Research Unit (MRC PHRU), Nuffield Department of Population Health, University of Oxford, Oxford, United Kingdom; ^7^Qingdao Center for Disease Control and Prevention, Qingdao, China; ^8^China National Center for Food Safety Risk Assessment, Beijing, China

**Keywords:** liver enzyme, fatty liver disease (FLD), cardiovascular disease, genetics, lifestyle

## Abstract

**Background and aims:**

Liver biomarkers and metabolic associated fatty liver disease (MAFLD) have been shown to be associated with cardiovascular disease (CVD). However, there is limited evidence on CVD subtypes [myocardial infarction (MI), ischemic stroke (IS), and intracerebral hemorrhage (ICH)], especially in the Chinese population. We examined these associations overall, by genetic predisposition to non-alcoholic fatty liver disease (NAFLD), and by lifestyle risk factors.

**Approach and results:**

This is a nested case-control study of CVD (10,298 cases and 5,388 controls) within the China Kadoorie Biobank. Cox regression was used to estimate adjusted hazard ratios (HRs) for CVD associated with liver biomarkers and MAFLD and by stratum of genetic risk and a combined high-risk lifestyle score. For liver enzymes, there were positive associations with MI and IS, but no associations with ICH or carotid plaque. There were positive associations of NAFLD with risks of MI, IS, and ICH (HR 1.43 [95% CI 1.30–1.57], 1.25 [1.16–1.35], and 1.12 [1.02–1.23]) as well as carotid plaque (odds ratio 2.36 [1.12–4.96]). The associations of NAFLD with CVD and carotid plaque were stronger among individuals with a high genetic risk (ICH: *p*-interaction < 0.05), while the associations with stroke were stronger among those with a favorable lifestyle (*p*-interaction < 0.05). The results for MAFLD mirrored those for NAFLD.

**Conclusion:**

In Chinese adults, liver biomarkers and MAFLD were associated with risk of CVD, with different magnitudes of associations by CVD subtypes. Genetic predisposition to NAFLD and lifestyle factors modified the associations of fatty liver with stroke.

## Introduction

Cardiovascular disease (CVD) is one of the leading causes of morbidity and mortality worldwide, especially in people over 50 years old (1). In China, there were 5.09 million deaths from CVD in 2019, with stroke and ischemic heart disease (IHD) ranking the top two causes of death (2). According to the Global Burden of Disease in 2019, the age-standardized incidence rate (ASIR) of stroke in China was much higher than that in Western countries, so was the proportion of intracerebral hemorrhage (ICH). Moreover, ICH and IS accounted for a similar number of deaths in China, despite a three-fold higher incidence of IS.

Several liver biomarkers, including liver enzymes and fatty liver index (FLI), have been reported to be associated with CVD (3–11). Serum levels of liver enzymes, including alanine aminotransferase (ALT), aspartate aminotransferase (AST), and γ-glutamyltransferase (GGT), are important indicators reflecting liver function and thus extensively measured in both clinical settings and population-based studies. FLI, calculated from triglycerides (TC), body mass index (BMI), GGT, and waist circumference (WC), is widely used as a non-invasive test for diagnosis of NAFLD in large-scale epidemiological studies (12). A recent meta-analysis reported that NAFLD was associated with increased risks of MI and IS (13). However, there is limited evidence on the associations of liver biomarkers with CVD subtypes (i.e., ischemic and hemorrhagic).

Previous prospective studies have shown a positive association between metabolic associated fatty liver disease (MAFLD), a newly suggested term to replace “non-alcoholic fatty liver disease (NAFLD)” (14), and CVD, both in Caucasians and East Asians (15–19). However, it is not clear whether the associations with CVD differ for NAFLD and MAFLD. Moreover, there is limited evidence whether the associations differ by genetic predisposition to NAFLD or lifestyle factors. Although a recent study in the UK Biobank (UKB) reported that fatty liver disease-related genetic variants amplified the health impact of MAFLD (16), the study was conducted in the Western population and may not be generalizable to the Chinese population, where substantial differences exist in genetics, lifestyles, and CVD subtypes. Assessing the associations between liver biomarkers and CVD may provide a better understanding of disease etiology and inform disease prognosis, while examining the interactions by genetic factors or lifestyles helps identify individuals who may benefit most from liver disease screening.

Therefore, we aimed to examine the associations of liver biomarkers (i.e., liver enzymes and FLI) and MAFLD with CVD subtypes and to assess whether the associations differ by genetic predisposition to NAFLD or lifestyle factors.

## Materials and methods

### Study population

Details of the CKB (China Kadoorie Biobank) prospective cohort study design, survey methods, population characteristics, and long-term follow-up have been described elsewhere (20). Briefly, 512,715 participants (male 41%) aged 30–79 years were recruited into the study from 10 (5 urban and 5 rural) diverse areas in China during 2004–2008. Central ethics approvals were obtained from the Chinese Center for Disease Control (CDC) and the University of Oxford as well as institutional research boards at the local CDCs in the 10 regions. Written informed consent were obtained from all participants. At local study assessment clinics, participants completed an interviewer-administered, laptop-based questionnaire on socio-demographic characteristics, smoking, alcohol consumption, diet, physical activity, personal and family medical histories, and current medication. A range of physical measurements were recorded by trained technicians, including height, weight, hip and WC, bio-impedance, lung function, blood pressure, and heart rate, using calibrated instruments with standard protocols. Detailed descriptions of data collection on lifestyle risk factors were shown in Supplementary Methods.

The present study excluded individuals with a prior history of cancer (*n* = 8), cirrhosis or hepatitis (*n* = 203), positive hepatitis B surface antigen (HBsAg) tests (*n* = 534), missing values of any liver biomarkers (*n* = 1,136), and without genotyping data (*n* = 616), leaving 15,686 individuals for the main analysis (Supplementary Figure 1).

### Nested case-control study of cardiovascular disease

The current analysis was based in a nested case-control study of CVD with clinical chemistry measurements among 18,183 participants. Cases were identified as those that had an incident fatal or non-fatal event coded as International Classification of Diseases, 10th Revision (ICD-10): I21-23 for myocardial infarction (MI, *n* = 1,273); I63 and I69.3 for ischemic stroke (IS, *n* = 5,447); I61 for ICH (*n* = 5,150) at the censoring date of 1 January 2015. Common controls (*n* = 6,313) were frequency matched to cases by age, sex, and region. Cases and controls were free of prior vascular disease (including absence of statin therapy) and cancer. Both MI and IS were atherosclerotic CVD, with etiologies differing from ICH. ICH cases were included to compare and contrast the associations between liver biomarkers and CVD subtypes.

### Assessment of liver biomarkers

Liver enzymes included ALT, AST, and GGT. Plasma concentrations of TC was measured by Beckman-Coulter AU680 clinical chemistry analyzers using manufacturers’ reagents, calibrators and settings (Beckman-Coulter, United Kingdom). FLI for each participant was calculated according to a previously published formula involving TC, BMI, GGT, and WC (12). FLI ≥60, without excessive drinking (≥30 g/day in men, ≥20 g/day in women) or concomitant liver diseases (i.e., viral hepatitis, alcoholic liver disease, toxic liver disease, biliary cholangitis, autoimmune hepatitis, Wilson’s disease, or hemochromatosis), was used as an indicator of NAFLD (12).

Metabolic associated fatty liver disease was diagnosed based on the FLI-defined hepatic steatosis (FLI ≥60) and the presence of any of the following three conditions: (1) overweight or obesity (BMI ≥23 kg/m^2^), (2) presence of type 2 diabetes mellitus (T2D), and (3) presence of at least 2 metabolic abnormalities, including increased WC (≥90/80 cm for men/women), arterial hypertension (≥130/85 mmHg or specific drug treatment), hypertriglyceridemia (≥150 mg/dl or ≥1.70 mmol/L or specific drug treatment), low high-density lipoprotein cholesterol (<40/50 mg/dl or <1.0/1.3 mmol/L for men/women or specific drug treatment), prediabetes (fasting glucose levels 100–125 mg/dl), and subclinical inflammation [plasma high-sensitivity C-reactive protein (CRP) level >2 mg/L]. Insulin resistance was not measured in CKB and thus excluded from our definition compared with the international expert consensus (21).

### Assessment of subclinical atherosclerosis

Detailed assessment of subclinical atherosclerosis was described in Supplementary Methods. In the current study, carotid plaque burden was used as the outcome measure of subclinical atherosclerosis. Carotid plaque burden was derived by standardizing the plaque number and maximum size [i.e., dividing each by its standard deviation (SD)] and estimating the average, then multiplying the average value by the SD of the maximum plaque thickness to provide a plaque burden recorded in millimeter units. Presence of carotid plaque was defined as carotid plaque burden ≥2.

### Genotyping measurement

A custom-designed 800K-single-nucleotide variation (SNV) array (Axiom, Affymetrix) with imputation to 1000 Genomes Phase 3 was utilized to conduct genotyping. Genotype data were available for samples from 100,408 participants passing quality controls (overall call rate >99.97% across all variants), which included a population-based sample of 75,736 participants randomly selected from the total CKB cohort and 24,672 participants genotyped as part of nested case-control studies. For the nested case-control study of clinical chemistry, 17,567 participants were genotyped.

*PNPLA3* p.I148M (rs738409), a well-established genetic variant for NAFLD in both Caucasians and East Asians (22), was selected as the target locus. The genotype was coded as 0, 1, and 2 for non-carriers, heterozygous carriers, and homozygous carriers of the risk-increasing allele, respectively.

### Definitions of high-risk lifestyle factors

We selected smoking, alcohol, physical activity, and central adiposity to construct a combined high-risk lifestyle score. These factors were selected because of their associations with risk of chronic liver disease in the Chinese population (23–25). To investigate the combined effects of high-risk lifestyle, we grouped each participant into 1 of 5 categories according to the number of healthy lifestyle factors (0–4), including smoking (current or former regular smokers), alcohol (weekly alcohol consumption ≥210 g, ex-regular or reduced-intake drinkers), physical inactivity [total physical activity <14.7 MET-h/day (the bottom 50%)], and central obesity [WC ≥90 cm (men) or 80 cm (women)]. The cut-off points were selected for each lifestyle factor based on *a priori* knowledge of the risk factors for chronic liver disease and are considered achievable at the population level.

### Statistical analysis

The primary outcome was CVD, and the secondary outcome was subclinical atherosclerosis. Cox proportional hazards regression models were used to calculate hazard ratios (HRs) of specific disease incidence and 95% confidence intervals (CIs), adjusted for age, sex, region, education, smoking, and alcohol. The underlying time scale was time since birth, and participants entered the study at their baseline age. We first examined the dose-response associations between liver biomarkers and incident CVD, using quintiles as cut-off points. Then, we examined the associations of genetic and high-risk lifestyle factors with incident CVD. To further examine whether the associations between liver biomarkers and CVD differed by genetic or lifestyle risk factors, we performed analyses stratified by genetic risk of NAFLD and the number of high-risk lifestyle factors, separately. Participants were divided into two groups according to their genetic predisposition (low risk, 0 alleles; high risk: 1–2 alleles) or the number of high-risk lifestyle factors (low risk, 0–1; high risk, 2–4). For carotid plaques, a logistic regression model was used to calculate odds ratios (ORs) and 95% CIs associated with liver biomarkers, adjusted for the same variables in the Cox regression.

To assess potentially non-linear associations between liver enzymes and CVD risk, restricted cubic splines were calculated using three fixed knots at the 10, 50, and 90% quintiles. Non-linearity was evaluated using the likelihood ratio rest to compare the fit of linear and non-linear models. Subgroup analyses were conducted by age (<60 vs. ≥60 years) and sex. In sensitivity analysis, we additionally adjusted for BMI and physical activity, potential risk factors for NAFLD.

For analyses involving more than two categories, all HRs are presented, with 95% CIs calculated using “floating” standard errors to facilitate comparisons between any two groups rather than just with the reference group (26). Instead of selecting 1 level of the risk factor as the reference group, a “floated” variance is assigned to each level, which describes the uncertainty in risk without reference to another level. All analyses were performed using R program (version 3.6.2; R Foundation for Statistical Computing, Vienna, Austria).

## Results

### Baseline characteristics of participants

Among the 15,686 participants, the mean age of ICH cases was similar to control subjects (Table 1), but the mean age of MI and IS cases was younger. Likewise, the proportion of women among ICH cases was similar to control subjects, but the proportion was lower among MI cases and higher among IS cases. Cases had higher SBP than control subjects, and they were more likely to have diabetes at baseline. Levels of physical activity, overall and central adiposity, and height were similar between cases and control subjects (Table 1).

**TABLE 1:** Baseline characteristics of participants by incident disease status.

	Controls	MI	IS	ICH
Variable	(*n* = 5,388)	(*n* = 1,170)	(*n* = 4,691)	(*n* = 4,437)
Age (SD), year	58.1 (11.1)	54.4 (8.7)	53.5 (9.5)	59.2 (10.5)
Female, %	2,566 (47.6)	472 (40.3)	2,547 (54.3)	2,142 (48.3)
**Socioeconomic and lifestyle factors**				
Urban region, %	1,197 (22.2)	396 (33.8)	2,066 (44.0)	977 (22.0)
≥9 years of education, %	703 (13.0)	181 (15.5)	1,086 (23.2)	433 (9.8)
Household income ≥35,000 RMB/year, %	524 (9.7)	134 (11.5)	662 (14.1)	412 (9.3)
Ever regular smoking, %				
Male	1,725 (61.1)	508 (72.8)	1,386 (64.6)	1,386 (60.4)
Female	88 (3.4)	23 (4.9)	69 (2.7)	88 (4.1)
Weekly drinking, %				
Male	819 (29.0)	195 (27.9)	724 (33.8)	679 (29.6)
Female	51 (2.0)	7 (1.5)	61 (2.4)	56 (2.6)
Total physical activity (SD), MET-h/day	19.0 (13.3)	19.3 (14.0)	18.6 (13.3)	17.6 (13.3)
Sedentary leisure time (SD), h/day	3.0 (1.5)	3.2 (1.5)	3.1 (1.5)	3.0 (1.6)
**Blood pressure and anthropometry**				
SBP (SD), mmHg	134.9 (21.1)	142.0 (25.4)	141.2 (25.2)	152.5 (27.6)
RPG (SD), mmol/L	6.0 (2.3)	6.9 (3.7)	6.6 (3.3)	6.5 (3.0)
BMI (SD), kg/m^2^	23.0 (3.3)	24.0 (3.7)	24.4 (3.5)	23.3 (3.6)
Waist circumference (SD), cm	79.2 (9.5)	83.0 (10.4)	83.0 (10.0)	80.5 (10.2)
Hip circumference (SD), cm	89.1 (6.7)	91.5 (7.5)	92.4 (7.0)	89.6 (7.0)
Waist-to-hip ratio (SD)	0.89 (0.07)	0.91 (0.07)	0.90 (0.07)	0.90 (0.07)
Percent body fat (SD), %	25.4 (8.7)	26.7 (8.9)	28.8 (8.7)	25.7 (9.1)
Height (SD), cm	158.3 (8.6)	160.7 (8.7)	159.5 (8.4)	157.3 (8.4)
**Liver biomarkers**				
NAFLD, %	489 (9.8)	205 (15.2)	709 (14.3)	518 (12.5)
MAFLD, %	619 (12.6)	265 (19.1)	917 (17.9)	645 (16.2)
AST (SD), μl	28.1 (13.4)	29.6 (26.8)	25.4 (1.06)	29.9 (19.4)
ALT (SD), μl	21.7 (15.6)	24.3 (19.1)	21.2 (14.3)	22.2 (16.8)
GGT (SD), μl	27.4 (42.4)	40.5 (90.7)	29.9 (41.5)	37.3 (107.1)
**Disease history**				
Diabetes, %	253 (5.0)	160 (13.3)	545 (11.0)	375 (8.7)

Values are mean ± SD or %.

MI, myocardial infarction; IS, ischemic stroke; ICH, intracerebral hemorrhage; BMI, body mass index; MET, metabolic equivalent of task; RPG, random plasma glucose; SBP, systolic blood pressure; FLI, fatty liver index; MAFLD, metabolic-associated fatty liver disease; AST, aspartate aminotransferase; ALT, alanine aminotransferase; GGT, γ-glutamyltransferase.

Overall, the proportions of NAFLD and MAFLD were similar in each group, and cases were more likely to meet the MAFLD criteria (MI 19.1%; IS 17.9%; ICH 16.2%; control subjects 2.6%). For liver enzymes, MI and ICH cases had higher mean concentrations of AST and ALT than control subjects, while IS cases had lower concentrations of AST than control subjects. Cases had higher concentrations of GGT than control subjects.

The average follow-up period of this study is 10 years.

### Associations of liver biomarkers with risk of cardiovascular disease

**FIGURE 1:**
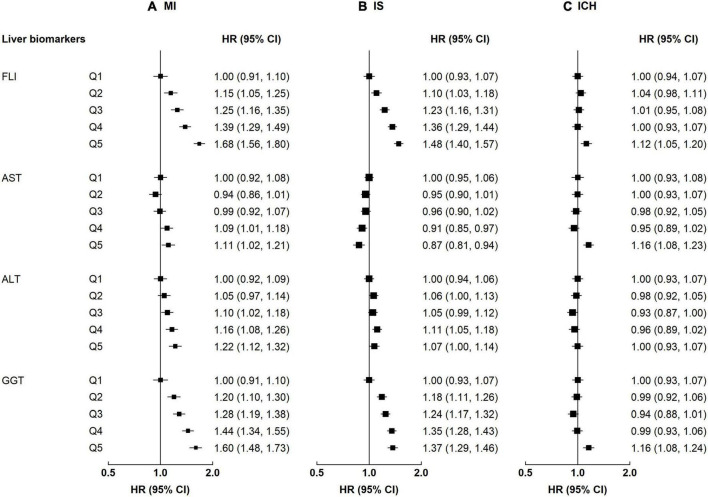
Associations of liver biomarkers with risk of CVD. Columns **(A–C)** denote the results for MI, IS, and ICH, respectively. Boxes represent the hazard ratios (HRs) of CVD associated with liver biomarkers, with the size of the box inversely proportional to the variance of the logHR. The model is adjusted for age, sex, regions, education, smoking, and alcohol.

There were positive associations of FLI and GGT with atherosclerosis cardiovascular disease (ASCVD) (i.e., MI and IS) but a weaker positive association with ICH. The adjusted HRs per 1-SD higher FLI were 1.17 (95% CI 1.13–1.21) for MI, 1.16 (1.13–1.19) for IS, and 1.04 (1.01–1.08) for ICH; the corresponding HRs for GGT were 1.08 (1.05, 1.11) for MI, 0.93 (0.90, 0.97) for IS and 1.10 (1.08, 1.12) for ICH (Supplementary Table 1). Of note, the positive association of FLI and GGT with ICH was only observed when comparing the top and the bottom quintile (FLI 1.12 [1.05–1.20] and GGT 1.16 [1.08, 1.24], Figure 1). Similar to FLI, there were positive associations between MAFLD and all CVD subtypes (MI 1.42 [1.30–1.55], IS 1.24 [1.15–1.33], and ICH 1.12 [1.03–1.22], Supplementary Table 3). Participants with high AST had higher risks of MI and ICH, but a lower risk of IS (HR comparing top vs. bottom quintile: MI 1.11 [1.02, 1.21], IS 0.87 [0.81, 0.94], and ICH 1.16 [1.08, 1.23]). For ALT, positive associations were observed with MI, but there were no associations with IS and ICH. Likelihood ratio tests showed evidence of significant non-linear associations of FLI with IS (*p*-value for non-linearity <0.01), ALT with IS and ICH (*p*-value for non-linearity ≤0.02), and GGT with MI and IS (*p*-value for non-linearity < 0.01) (Supplementary Figure 3).

When additionally adjusting for BMI and physical activity, the associations of liver biomarkers with CVD slightly attenuated, but the patterns remained (Supplementary Figure 2). The associations of FLI and GGT with ICH differed by sex (*p*-value for interaction ≤0.02), while the associations of ALT and GGT with MI differed by age (*p*-value for interaction ≤0.01, Supplementary Table 4). For example, FLI was positively associated with ICH among men, but no such association was observed in women. For MI, the associations with ALT and GGT attenuated among those ≥60 years (Supplementary Figure 4).

### Associations of high-risk lifestyle and genetic risk factors with risk of cardiovascular disease

**FIGURE 2:**
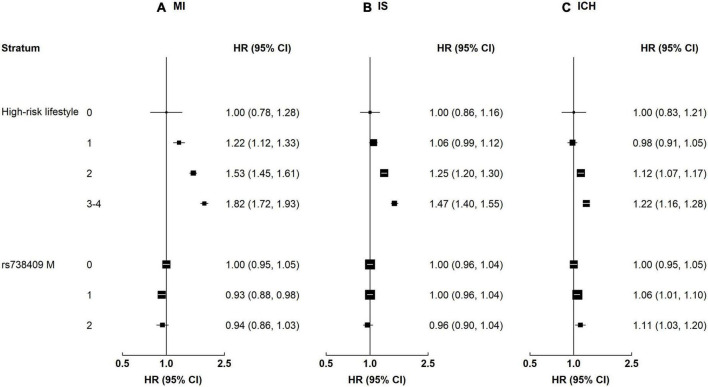
Associations of high-risk lifestyle and genetic risk factors with risk of CVD. Columns **(A–C)** denote the results for MI, IS, and ICH, respectively. Convention as in Figure 1.

Alcohol and central adiposity were each associated with higher risks of all CVD subtypes (Supplementary Table 2). The HRs for alcohol were 1.12 (1.04–1.21) for MI, 1.13 (1.07–1.20) for IS, and 1.18 (1.10–1.26) for ICH. The HRs for central adiposity were 1.38 (1.28–1.48) for MI, 1.34 (1.27–1.42) for IS, and 1.08 (1.00–1.17) for ICH. Smoking was associated with a higher risk of MI (1.27 [1.15–1.41]), and the positive association was borderline significant for IS (1.07 [0.99–1.17]). Physical inactivity was associated with ICH (1.08 [1.00–1.17]). When combining these four high-risk lifestyle factors, there were positive associations between the number of lifestyle factors and risk of all CVD subtypes (Figure 2). Compared with those without high-risk lifestyle factors, the HRs for MI were 1.22 (1.12–1.33), 1.53 (1.45–1.61), and 1.82 (1.72–1.93) among participants with 1, 2, and 3–4 lifestyle factors, respectively. The corresponding HRs for IS were 1.06 (0.99–1.12), 1.25 (1.20–1.30), and 1.47 (1.40–1.55), while the corresponding HRs for ICH were 0.98 (0.91–1.05), 1.12 (1.07–1.17), and 1.22 (1.16–1.28). For rs738409, there was a positive association between the number of rs738409 M allele with ICH, but no association was observed for MI or IS. Compared with those with 0 risk alleles, the HRs for ICH were 1.06 (1.01–1.10) and 1.11 (1.03–1.20) among participants with 1 allele and 2 alleles, respectively.

### Associations of liver biomarkers with risk of cardiovascular disease by genetic predisposition to non-alcoholic fatty liver disease

**FIGURE 3:**
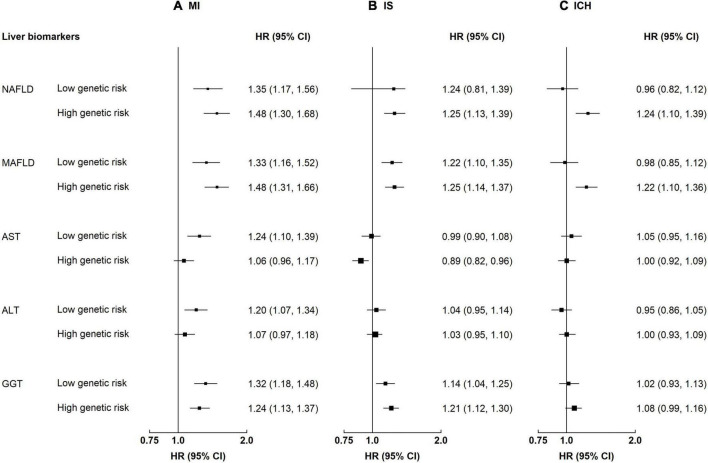
Associations of liver biomarkers with risk of CVD by genetic predisposition to NAFLD. Columns **(A–C)** denote the results for MI, IS, and ICH, respectively. Boxes represent the hazard ratios (HRs) of CVD associated with high liver biomarkers, with the size of the box inversely proportional to the variance of the logHR. Liver biomarker was each modeled as a dichotomous variable. The cut-off points are: ≥median for AST, ALT, and GGT. High genetic risk denotes 1–2 risk increasing alleles of rs73409. Low genetic risk denotes 0 risk increasing alleles of rs73409. The model is adjusted for age, sex, regions, education, smoking, and alcohol. *p*-Values for interaction: MI 0.11–0.50, IS 0.05–0.81, and ICH 0.009–0.54. *p*-Values for interaction <0.05: ICH, FLI 0.009, MAFLD 0.01.

Participants with NAFLD or MAFLD had higher risks of MI and IS, regardless of the genetic risk (*p*-value for interaction 0.21–0.77, Supplementary Table 3). Although the interaction by genetic predisposition to NAFLD was non-significant, the HRs tended to be stronger among individuals with a high genetic risk (Figure 3). For instance, the HRs associated with MAFLD among participants with a low genetic risk were 1.33 (1.16–1.52) for MI and 1.22 (1.10–1.35) for IS, while the HRs among those with a high genetic risk were 1.48 (1.31–1.66) and 1.25 (1.14–1.37). In contrast, the associations of NAFLD and MAFLD with ICH differed by genetic predisposition to NAFLD, with stronger associations among those with a high genetic risk. For NAFLD, the HRs were 0.96 (0.82–1.12) and 1.24 (1.10–1.39) among those with a low and a high genetic risk, respectively; the corresponding HRs for MAFLD were 0.98 (0.85–1.12) and 1.22 (1.10–1.36) (for both NAFLD and MAFLD, *p*-value for interaction <0.05). Genetic predisposition to NAFLD did not interact with liver enzymes on risk of CVD (*p*-value for interaction 0.05–0.78).

### Associations of liver biomarkers with risk of cardiovascular disease by lifestyle factors

**FIGURE 4:**
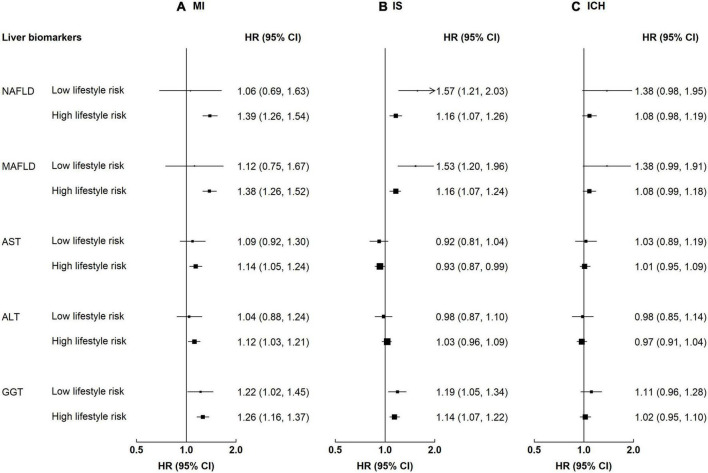
Associations of liver biomarkers with risk of CVD by lifestyle factors. Columns **(A–C)** denote the results for MI, IS, and ICH, respectively. Convention as in Figure 3. Low lifestyle risk denotes 0–1 high-risk lifestyle factors. High lifestyle risk denotes 2–4 high-risk lifestyle factors. *p*-Values for interaction: MI 0.28–0.82, IS 0.02–0.74, and ICH 0.04–0.61. *p*-Values for interaction <0.05: IS, NAFLD 0.03, MAFLD 0.02; ICH, FLI 0.04, MAFLD 0.04.

Participants with NAFLD or MAFLD had higher risks for MI, regardless of the lifestyle risk (*p*-value for interaction 0.28–0.35, Supplementary Table 3). The HR associated with MAFLD among participants with a low lifestyle risk was 1.12 (0.75–1.67) for MI, while the HR among those with a high lifestyle risk was 1.38 (1.26–1.52). In contrast, the associations of NAFLD and MAFLD with IS and ICH differed by lifestyle factors (Figure 4). For NAFLD, the HRs of IS were 1.57 (1.21–2.03) and 1.16 (1.07–1.26) among those with a low and a high lifestyle risk, respectively; the corresponding HRs of ICH were 1.38 (0.98–1.95) and 1.08 (0.98–1.19) (for both NAFLD and MAFLD, *p*-value for interaction <0.05). Lifestyle risk factors did not interact with liver enzymes on risk of CVD (*p*-value for interaction 0.19–0.82).

### Associations of liver biomarkers with subclinical atherosclerosis

Overall, there were positive associations of NAFLD and MAFLD with of carotid plaque and no associations were observed for liver enzymes (Supplementary Table 5). For both NAFLD and MAFLD, the associations tended to be stronger among individuals with a high genetic risk, though the interaction was non-significant (*p*-value for interaction 0.18). Similar associations were observed when stratified by high-risk lifestyle factors.

## Discussion

This study provides a comprehensive examination of the associations of liver biomarkers with risk of CVD by subtypes. The results showed that there were positive associations of liver enzymes with ASCVD (i.e., MI and IS) and weaker associations for ICH, regardless of the genetic predisposition to NAFLD or lifestyle risk factors. Participants with NAFLD or MAFLD had higher risks of all CVD subtypes as well as subclinical atherosclerosis, with stronger associations among individuals with a high genetic risk of NAFLD. For both IS and ICH, the associations of NAFLD and MAFLD were stronger among individuals with a favorable lifestyle.

The current study findings for liver biomarkers were generally consistent with previous studies examining the associations of liver enzymes in relation to CVD (4, 6, 8, 9, 27–29). These studies include a meta-analysis published in 2019 involving 1,067,922 participants and a prospective cohort study involving 416,122 participants in Taiwan (3, 6), both examining CVD mortality. For MI and IS, our findings for ALT and GGT were similar to the associations reported by two large cohort studies involving 6,912,393 participants in Korea (4, 9). In contrast to our null findings for ICH, a cohort of 108,464 Korean men showed positive associations of AST and ALT with ICH (28). For stroke subtypes, two cohorts conducted in Western countries reported positive associations of ALT and AST with ICH (8, 29), while one United States cohort reported a positive association between GGT and IS (8). However, previous studies involved a small number of ICH cases (ranging from 90 to 718) and the associations may be due to chance. Although the current study included a large number of CVD subtypes (5,447 IS cases and 5,150 ICH cases), future studies are still warranted to examine the associations of liver enzymes with CVD subtypes.

Fatty liver index is a commonly used indicator of NAFLD in large population-based studies. Recently, an expert consensus recommended the change from NAFLD to MAFLD, since the latter included more metabolic factors and thus might be more sensitive in demonstrating disease progression (14, 16). Therefore, we explored the associations of MAFLD in relation to CVD as a complement to NAFLD. Notably, our study findings for NAFLD and MAFLD were fairly close, due to the fact that FLI ≥60 captured individuals who met the MAFLD definition in CKB. Previous studies have shown that both NAFLD and MAFLD were associated with higher risk of CVD (7, 10, 11, 13, 16, 18, 19, 30), particularly MI and IS, which was in line with our findings. For intermediate phenotypes, our findings of positive associations of NAFLD and MAFLD with subclinical atherosclerosis were also consistent with previous studies (31, 32). However, only a limited number of studies have examined the association of NAFLD or MAFLD with hemorrhagic CVD and further compared it with the associations with ischemic CVD. Our study filled this gap and showed that compared to ischemic CVD, there were weaker associations of NAFLD and MAFLD with ICH, which indicated that the prognostic role of NAFLD and MAFLD was more important for ischemic CVD.

There is limited evidence whether the associations between liver biomarkers and CVD differ by genetic or lifestyle risk factors. A recent study conducted in the UKB showed that the adverse impact of MAFLD on CVD (mainly ASCVD) was somewhat larger in individuals with a high genetic risk of NAFLD compared with those with a low genetic risk (low 1.37 [1.30–1.44], high 1.42 [1.33–1.50]) (16). This study finding was generally consistent with the results of MI and IS in CKB. In addition to ASCVD, we showed that the associations of MAFLD and NAFLD with ICH were much stronger among individuals with a high genetic risk of NAFLD. The effect modification by genetic risk was only observed for ICH, suggesting that the genetic variants may have little contribution to the development of ischemic CVD compared with hemorrhagic CVD. This is possibly because that high genetic risk amplifies the impact of metabolic factors on the progression of hypertension, a risk factor showing a stronger association with ICH than ischemic CVD in Chinese (33). Regarding lifestyle risk factors, we showed that the associations of MAFLD and NAFLD with both IS and ICH were stronger among individuals with a low-risk lifestyle. A probable explanation was that individuals with a favorable lifestyle differed in other risk factors for stroke (e.g., variability of blood pressure) (34, 35). Future studies in independent cohorts are needed to replicate our findings.

Our study has several clinical implications. Levels of liver biomarkers not only reflect the status of liver function, but also play an important role in CVD prediction, as demonstrated by our and previous studies. Since liver biomarkers are relatively easy to measure, these can be routinely monitored in clinical practice to identify patients at risk of CVD. Our results showed positive associations for both FLI and MAFLD with CVD risk regardless of the subtypes, suggesting that these two may be better indicators for CVD than liver enzymes. Regarding genetic risk stratification, although effect modification was only observed for ICH, the associations of FLI and MAFLD with CVD were both stronger among individuals with a high genetic risk of NAFLD. This suggests that FLI monitoring and MAFLD screening may be particularly important for those with genetic predisposition to NAFLD to predict risk of CVD.

The strengths of the CKB included a prospective design, a large study population, detailed adjustments for risk factors of CVD, and ascertainment of CVD through linkage to hospital records in addition to death and cancer registries. However, our study also had several limitations. First, we used FLI rather than imaging or biopsy to diagnose NAFLD, which was not the gold standard (36, 37). However, FLI has been externally validated in population-based studies conducted in Western countries and in China and is accepted by clinical practice guidelines as a proxy for imaging or biopsy in large-scale epidemiological studies (38–42). Second, insulin resistance, as a criterion of MAFLD diagnosis, was not assessed in our study due to the lack of serum insulin data in CKB. However, these two limitations might result in non-differential misclassification of NAFLD and MAFLD cases, and thus underestimating the associations between NAFLD/MAFLD and CVD. Third, it is probable that components of genetic predisposition to NAFLD other than rs738409 were not included. However, rs738409 had been recognized as the most important genetic variant of NAFLD across ethnicities (22). A meta-analysis of 12 Asian studies (7 of which were Chinese) with 4,495 cases reported that rs738409 M allele carriers were nearly twice as likely to develop NAFLD as non-carriers (OR 1.92 [1.54–2.39]) (43). Finally, residual confounding due to unmeasured or unknown variables cannot be ruled out (e.g., liver disease medications).

## Conclusion

In conclusion, this study in a Chinese population showed that liver biomarkers were associated with risk of CVD, with different magnitudes of associations by CVD subtypes. Genetic predisposition to NAFLD and lifestyle factors modified the associations of fatty liver with stroke, particularly ICH. The current study findings may inform CVD risk stratification using liver biomarkers in Chinese. FLI monitoring and MAFLD screening may be recommended among those with a high genetic risk of NAFLD.

## Data availability statement

Data described in the article, code book, and analytic code will be made available from the China Kadoorie Biobank upon request (http://www.ckbiobank.org/site/Data+Access), pending application and approval. The datasets generated and/or analyzed during the current study are not publicly available but are available from the corresponding author on reasonable request.

## Ethics statement

The studies involving human participants were reviewed and approved by the Chinese Center for Disease Control (CDC) and the University of Oxford as well as institutional research boards at the local CDCs in the 10 regions. The patients/participants provided their written informed consent to participate in this study.

## Author contributions

LL and ZC had full access to the data. XW, SC, YP, and LL conducted data analysis and were responsible for accuracy of the results and the decision to submit for publication. All authors were involved in study design, conduct, long-term follow-up, review and coding of disease events, interpretation of the results, or writing the report, and read and approved the final version of the manuscript.

## Abbreviations

ALT: alanine aminotransferase
ASCVD: atherosclerosis cardiovascular disease
AST: aspartate aminotransferase
ASIR: age-standardized incidence rate
BMI: body mass index
CDC: Chinese Center for Disease Control
CFR: case fatality rate
CI: confidence interval
CKB: China Kadoorie Biobank
CRP: C-reactive protein
CVD: cardiovascular disease
FLI: fatty liver index
GGT: γ-glutamyltransferase
HBsAg: hepatitis B surface antigen
HR: hazards ratio
ICD-10: International Classification of Diseases, 10th Revision
ICH: intracerebral hemorrhage
IHD: ischemic heart disease
IS: ischemic stroke
MAFLD: metabolic associated fatty liver disease
MI: myocardial infarction
NAFLD: non-alcoholic fatty liver disease
SD: standard deviation
SNV: single-nucleotide variation
OR: odds ratio
TC: triglycerides
T2D: type 2 diabetes mellitus
UKB: UK Biobank
WC: waist circumference
