# Supplementary material for: Liver biomarkers, genetic and lifestyle risk factors in relation to risk of cardiovascular disease in Chinese
Source: Front Cardiovasc Med. 2022 Aug 11;9:938902. doi: 10.3389/fcvm.2022.938902 (PMC9403237; doi:10.3389/fcvm.2022.938902)
Supplement: Supplementary file 1 [file Data_Sheet_1.docx]

**On-line Supplementary Material**

**Liver biomarkers, genetic and lifestyle risk factors in relation to risk of cardiovascular disease in Chinese**

**Frontiers in Cardiovascular Medicine**

Xinyu Wang, BM^1^*, Si Cheng, BM^1^*, Jun Lv, PhD^1,2,3^, Canqing Yu, PhD^1,2^, Yu Guo, MSc^4^, Pei Pei, MSc^4^, Ling Yang, PhD^5,6^, Iona Y. Millwood, DPhil^5,6^, Robin Walters, PhD^5,6^, Yiping Chen, DPhil^5,6^, Huaidong Du, PhD^5,6^, Haiping Duan, PhD^7^, Simon Gilbert, MSc^5^, Daniel Avery, MSc^5^, Junshi Chen, MD^8^, Yuanjie Pang, DPhil^1^, Zhengming Chen, DPhil^5,6^, Liming Li, MD^1,2^, on behalf of the China Kadoorie Biobank Collaborative Group†

*Joint first author

†The members of steering committee and collaborative group are listed in the supplemental material

1. Department of Epidemiology & Biostatistics, School of Public Health, Peking University, 38 Xueyuan Road, Beijing 100191, China
2. Peking University Center for Public Health and Epidemic Preparedness & Response, 38 Xueyuan Road, Beijing 100191, China
3. Key Laboratory of Molecular Cardiovascular Sciences (Peking University), Ministry of Education, Beijing, China
4. Chinese Academy of Medical Sciences, 9 Dongdan San Tiao, Beijing 100730, China
5. Clinical Trial Service Unit & Epidemiological Studies Unit (CTSU), Nuffield Department of Population Health, Big Data Institute Building, Roosevelt Drive, University of Oxford, UK
6. Medical Research Council Population Health Research Unit (MRC PHRU) at the University of Oxford, Nuffield Department of Population Health, University of Oxford, UK
7. Qingdao Center for Disease Control and Prevention, 175 Shandong Road, Qingdao 266033, China
8. National Center for Food Safety Risk Assessment, 37 Guangqu Road, Beijing 100021, China

**Address for correspondence:**

| Dr. Yuanjie Pang | Prof. Liming Li |
| --- | --- |
| Dept. of Epidemiology and Biostatistics | Dept. of Epidemiology and Biostatistics |
| School of Public Health | School of Public Health |
| Peking University | Peking University |
| 38 Xueyuan Road | 38 Xueyuan Road |
| Beijing, 100191, China | Beijing, 100191, China |
| Tel: 86-010-82801528 | Tel: 86-010-82801528 |
| Fax: 86-010-82801530 | Fax: 86-010-82801530 |
| yuanjie_p@163.com | lmleeph@vip.163.com |

**Table of content**

[Members of the China Kadoorie Biobank collaborative group 3](#_Toc109909154)

[Supplementary methods 5](#_Toc109909155)

[Supplementary Figure 1. Flow chart of study population for CVD and atherosclerosis analyses 8](#_Toc109909156)

[Supplementary Figure 2. Associations of liver biomarkers with risk of CVD additionally adjusting for BMI and physical activity 9](#_Toc109909157)

[Supplementary Figure 3. Non-linear associations of liver biomarkers with risk of CVD 10](#_Toc109909158)

[Supplementary Figure 4. Associations of liver biomarkers with risk of CVD by age and sex 11](#_Toc109909159)

[Supplementary Table 1. Associations of liver biomarkers with risk of CVD 14](#_Toc109909160)

[Supplementary Table 2. Associations of individual high-risk lifestyle risk factors with risk of CVD 15](#_Toc109909161)

[Supplementary Table 3. Associations of liver biomarkers with risk of CVD 16](#_Toc109909162)

[Supplementary Table 4. *P*-values for interaction by sex and age 18](#_Toc109909163)

[Supplementary Table 5. Associations of liver biomarkers with carotid plaque 19](#_Toc109909164)

# Members of the China Kadoorie Biobank collaborative group

**International Steering Committee:** Junshi Chen, Zhengming Chen (PI), Robert Clarke, Rory Collins, Yu Guo, Liming Li (PI), Jun Lv, Richard Peto, Robin Walters. **International Co-ordinating Centre, Oxford:** Daniel Avery, Ruth Boxall, Derrick Bennett, Yumei Chang, Yiping Chen, Zhengming Chen, Robert Clarke, Huaidong Du, Simon Gilbert, Alex Hacker, Mike Hill, Michael Holmes, Andri Iona, Christiana Kartsonaki, Rene Kerosi, Ling Kong, Om Kurmi, Garry Lancaster, Sarah Lewington, Kuang Lin, John McDonnell, Iona Millwood, Qunhua Nie, Jayakrishnan Radhakrishnan, Paul Ryder, Sam Sansome, Dan Schmidt, Paul Sherliker, Rajani Sohoni, Becky Stevens, Iain Turnbull, Robin Walters, Jenny Wang, Lin Wang, Neil Wright, Ling Yang, Xiaoming Yang. **National Co-ordinating Centre, Beijing:** Yu Guo, Xiao Han, Can Hou, Jun Lv, Pei Pei, Chao Liu, Canqing Yu. **10 Regional Co-ordinating Centres: Qingdao CDC:** Zengchang Pang, Ruqin Gao, Shanpeng Li, Shaojie Wang, Yongmei Liu, Ranran Du, Yajing Zang, Liang Cheng, Xiaocao Tian, Hua Zhang, Yaoming Zhai, Feng Ning, Xiaohui Sun, Feifei Li. **Licang CDC:** Silu Lv, Junzheng Wang, Wei Hou. **Heilongjiang Provincial CDC:** Mingyuan Zeng, Ge Jiang, Xue Zhou. **Nangang CDC:** Liqiu Yang, Hui He, Bo Yu, Yanjie Li, Qinai Xu,Quan Kang, Ziyan Guo. **Hainan Provincial CDC:** Dan Wang, Ximin Hu, Jinyan Chen, Yan Fu, Zhenwang Fu, Xiaohuan Wang. **Meilan CDC:** Min Weng, Zhendong Guo, Shukuan Wu,Yilei Li, Huimei Li, Zhifang Fu. **Jiangsu Provincial CDC:** Ming Wu, Yonglin Zhou, Jinyi Zhou, Ran Tao, Jie Yang, Jian Su. **Suzhou CDC:** Fang liu, Jun Zhang, Yihe Hu, Yan Lu, Liangcai Ma, Aiyu Tang, Shuo Zhang, Jianrong Jin, Jingchao Liu. **Guangxi Provincial CDC:** Zhenzhu Tang, Naying Chen, Ying Huang. **Liuzhou CDC:** Mingqiang Li, Jinhuai Meng, Rong Pan, Qilian Jiang, Jian Lan,Yun Liu, Liuping Wei, Liyuan Zhou, Ningyu Chen Ping Wang, Fanwen Meng, Yulu Qin,, Sisi Wang. **Sichuan Provincial CDC:** Xianping Wu, Ningmei Zhang, Xiaofang Chen, Weiwei Zhou. **Pengzhou CDC:** Guojin Luo, Jianguo Li, Xiaofang Chen, Xunfu Zhong, Jiaqiu Liu, Qiang Sun. **Gansu Provincial CDC:** Pengfei Ge, Xiaolan Ren, Caixia Dong. **Maiji CDC:** Hui Zhang, Enke Mao, Xiaoping Wang, Tao Wang, Xi Zhang. **Henan Provincial CDC:** Ding Zhang, Gang Zhou, Shixian Feng, Liang Chang, Lei Fan. **Huixian CDC:** Yulian Gao, Tianyou He, Huarong Sun, Pan He, Chen Hu, Xukui Zhang, Huifang Wu, Pan He. **Zhejiang Provincial CDC:** Min Yu, Ruying Hu, Hao Wang. **Tongxiang CDC:** Yijian Qian, Chunmei Wang, Kaixu Xie, Lingli Chen, Yidan Zhang, Dongxia Pan, Qijun Gu. **Hunan Provincial CDC:** Yuelong Huang, Biyun Chen, Li Yin, Huilin Liu, Zhongxi Fu, Qiaohua Xu. **Liuyang CDC:** Xin Xu, Hao Zhang, Huajun Long, Xianzhi Li, Libo Zhang, Zhe Qiu.

# Supplementary methods

*Data collection on lifestyle risk factors*

All anthropometric measurements were taken to the nearest 0.1 cm or 0.1 kg by trained staff. Weight was measured with a body composition analyzer (TANITA-TBF-300GS, Tanita Corporation), subtracting the weight of clothing according to season (0.5kg in summer and 2.0-2.5kg in winter). BMI was calculated as weight (in kilograms) divided by the square of height (in meters). WC was measured using a soft, non-stretchable tape.

For smoking, the questionnaire covered frequency, duration, amount, and type of tobacco, as well as the ages at which participants began smoking regularly and ceased smoking, and the main reasons for stopping. Smoking status was categorized as (1) never (not smoking at baseline and had smoked <100 cigarettes in lifetime), (2) occasional (neither never nor former smokers and had not stopped smoking completely for at least the 6 months before baseline), (3) former regular (had smoked ≥100 cigarettes but had quit smoking by choice for ≥6 months before baseline), or (4) current regular smoker (ever smoked ≥1 cigarettes daily for ≥6 months).

For alcohol drinking, the questionnaire covered frequency, the type (beer, wine or spirits) and amount of each type consumed in a typical drinking week, as well as the age at which participants started drinking. Drinking status was classified into five categories as (1) abstainers (never had drunk alcohol in the past year and had not drunk weekly in the past), (2) occasional drinkers (had drunk alcohol occasionally, monthly but less than weekly, or during certain seasons, and had not drunk weekly in the past), (3) reduced-intake drinkers (had drunk alcohol occasionally, monthly but less than weekly, or during certain seasons, but had drunk weekly in the past), (4) ex-weekly drinkers (never had drunk alcohol in the past year but had drunk weekly in the past), or (5) weekly drinkers (often drank at least weekly during the past year).

At baseline and subsequent resurveys, participants were asked about the frequency, duration and type (intensity) of physical activity in four domains (i.e., occupation, commuting, housework and leisure-time exercise) during the past year. To quantify the amount of physical activity, metabolic equivalent of tasks (MET) from the 2011 update of compendium of physical activities was used. The MET value for a particular type of physical activity represents the ratio of the energy expended per kilogram of body weight per hour during that activity relative to that expended when sitting quietly. The number of hours spent per day participating in each activity was multiplied by the MET value for that activity, and the daily amount of total physical activity was obtained by summing the MET-hours/day for activities related to occupational and non-occupational (i.e., commuting, housework and non-sedentary leisure time) activities. Hours spent per day on sedentary leisure-time activities (such as television watching, reading, and playing cards or mahjong) and sleeping were also recorded, but were not included in the physical activity calculation.

*Assessment of subclinical atherosclerosis*

All participants had a carotid ultrasound examination conducted on a single occasion. Longitudinal scanning of the entire length of the carotid arteries (from the base of the neck to the angle of the jaw) was conducted bilaterally to screen for pre-plaques and plaques in each of the four segments of the carotid arteries. Consistent with the Manheim consensus(1), a pre-plaque was defined as any focal thickening of carotid intima-media thickness (CIMT) > 1.0 and ≤1.5 mm, and a plaque was defined as any focal thickening or protrusion from the wall into the lumen with CIMT >1.5 mm thickness. The number and location (i.e. segment) of pre-plaques and plaques were recorded by the sonographers. Cross-sectional scanning of the carotid arteries was used to record the thickness of the largest plaque or pre-plaque. Random samples (~3%) of carotid ultrasound examinations were checked for quality assessment by one of three radiologists who confirmed that CIMT had been measured correctly at four common carotid artery (CCA) segments, and that presence and number of plaques and the thickness of the maximum plaque had been located and counted satisfactorily in each of the 10 CCA segments.

**References**

1. Stein JH, Korcarz CE, Hurst RT, Lonn E, Kendall CB, Mohler ER, et al. Use of carotid ultrasound to identify subclinical vascular disease and evaluate cardiovascular disease risk: a consensus statement from the American Society of Echocardiography Carotid Intima-Media Thickness Task Force. *J Am Soc Echocardiogr*. 2008;21(2):93-111.

# Supplementary Figure 1. Flow chart of study population for CVD and atherosclerosis analyses


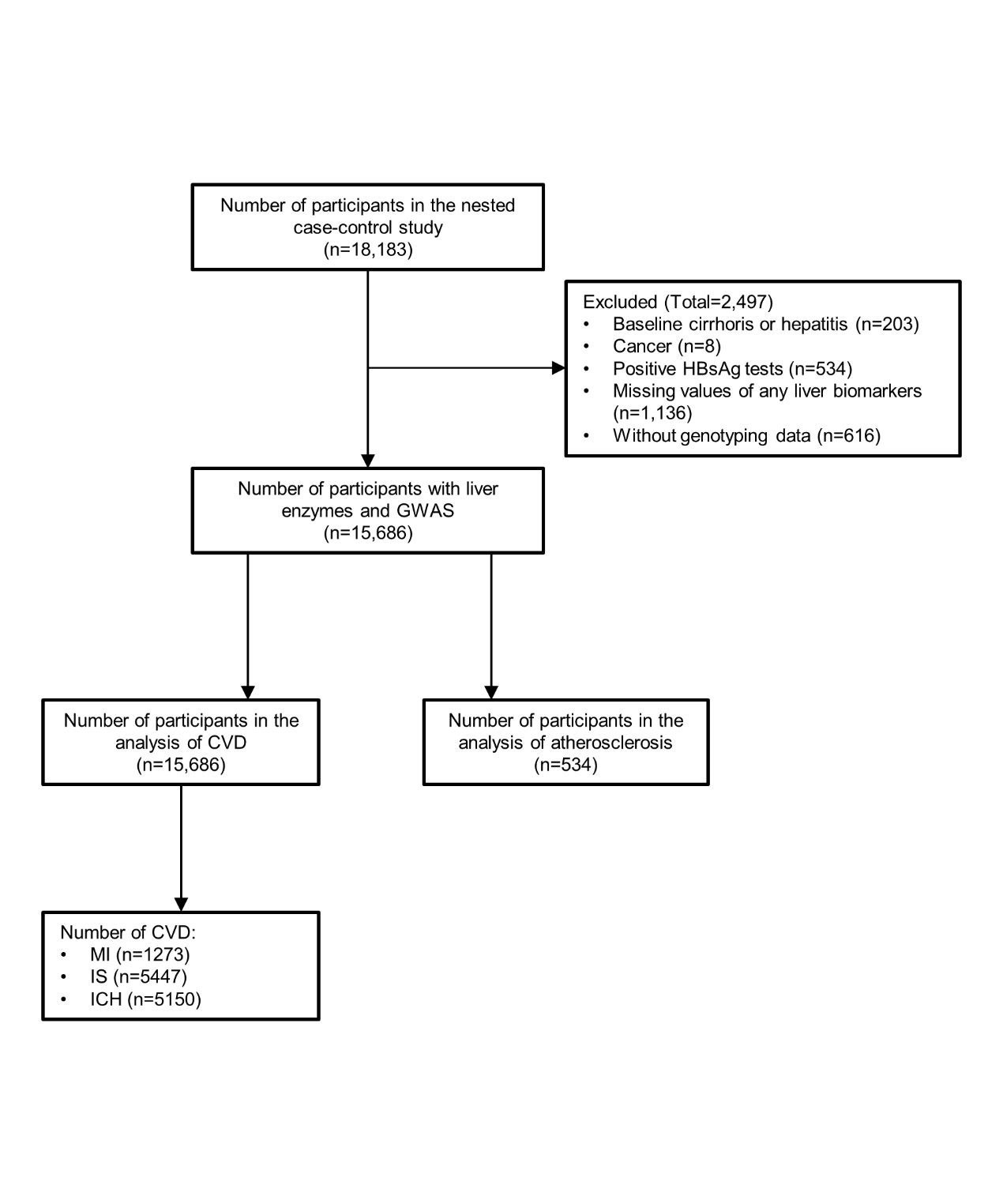


# Supplementary Figure 2. Associations of liver biomarkers with risk of CVD additionally adjusting for BMI and physical activity

**
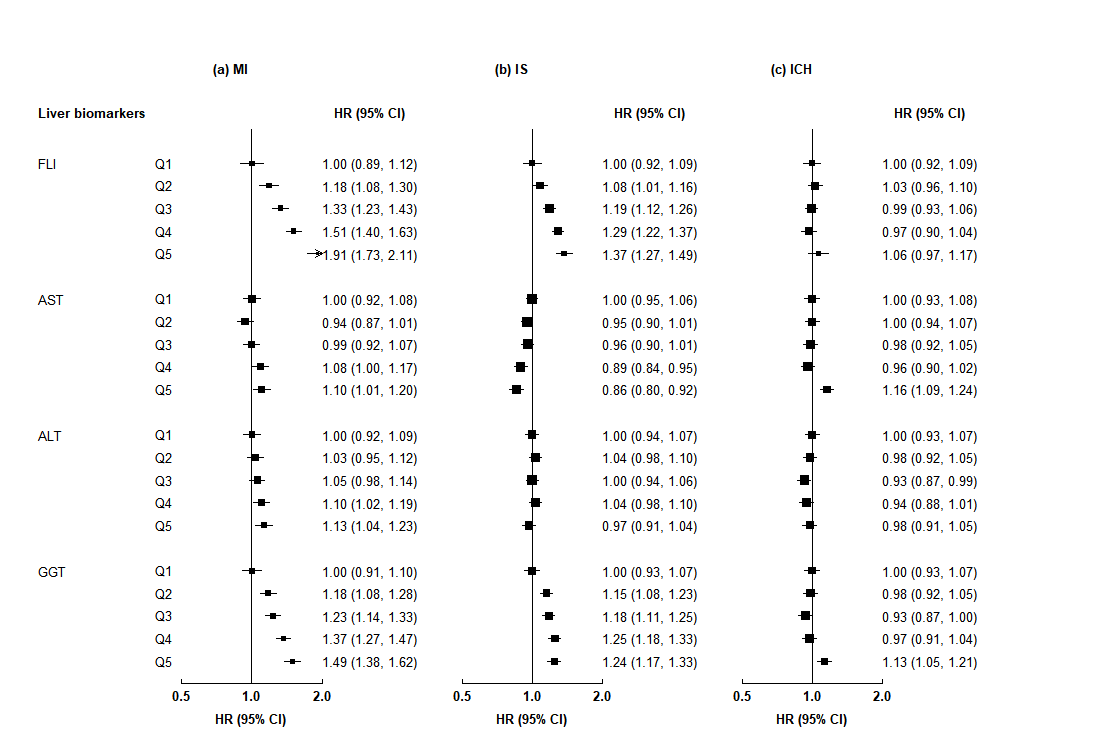
**

# Supplementary Figure 3. Non-linear associations of liver biomarkers with risk of CVD


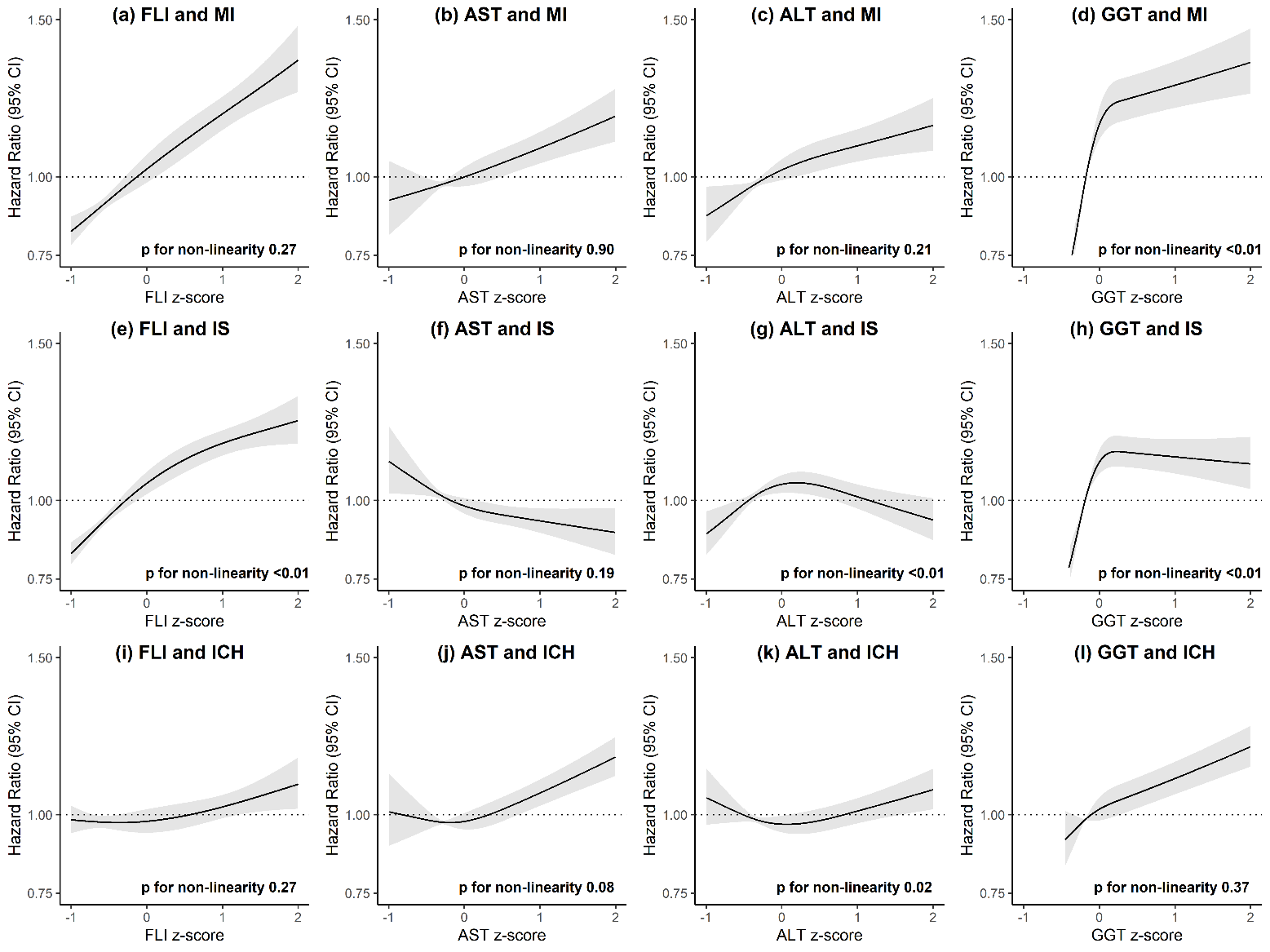


# Supplementary Figure 4. Associations of liver biomarkers with risk of CVD by age and sex

**
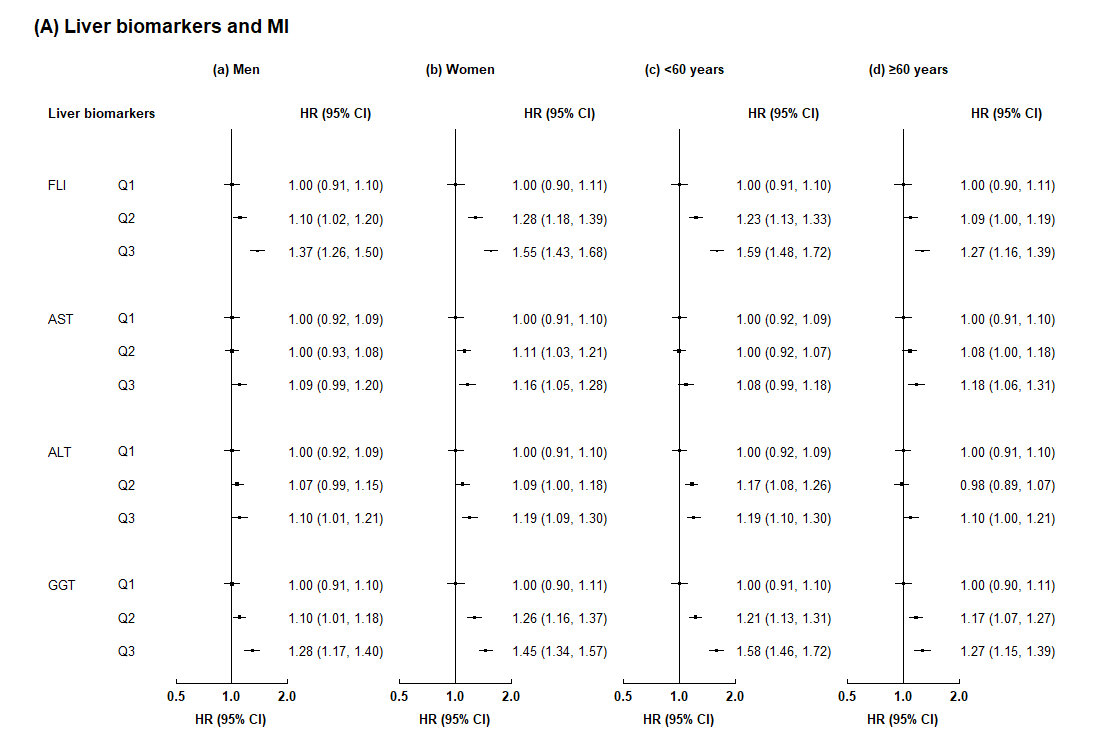
**

**
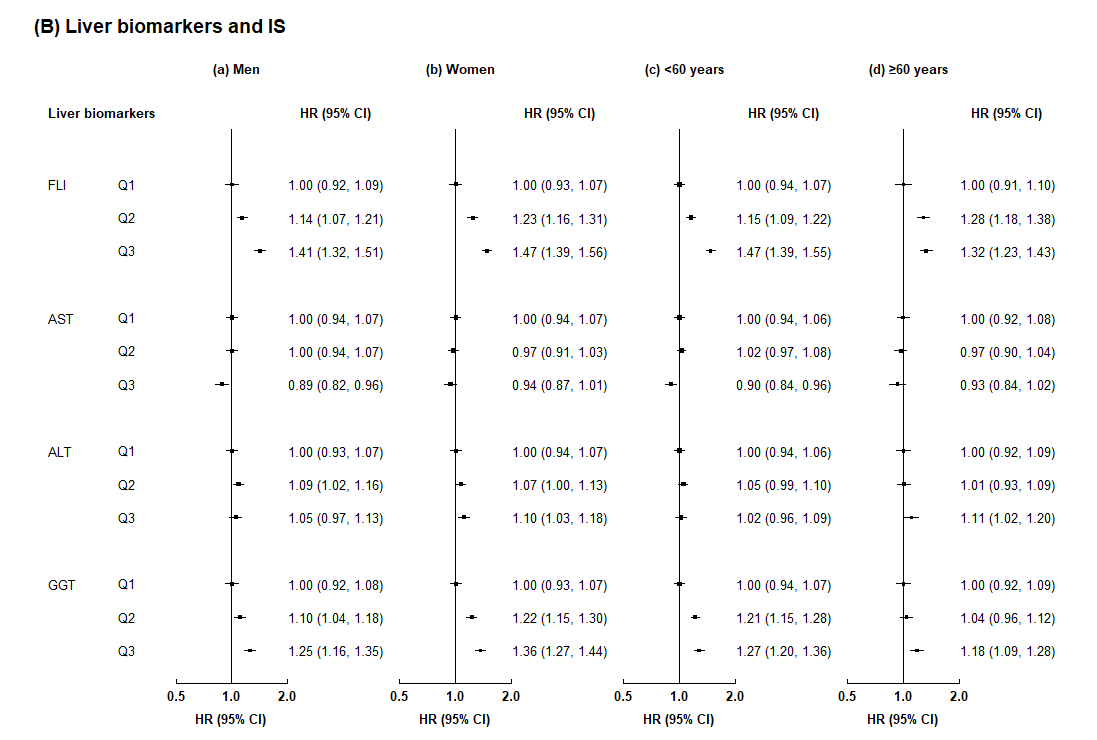
**

**
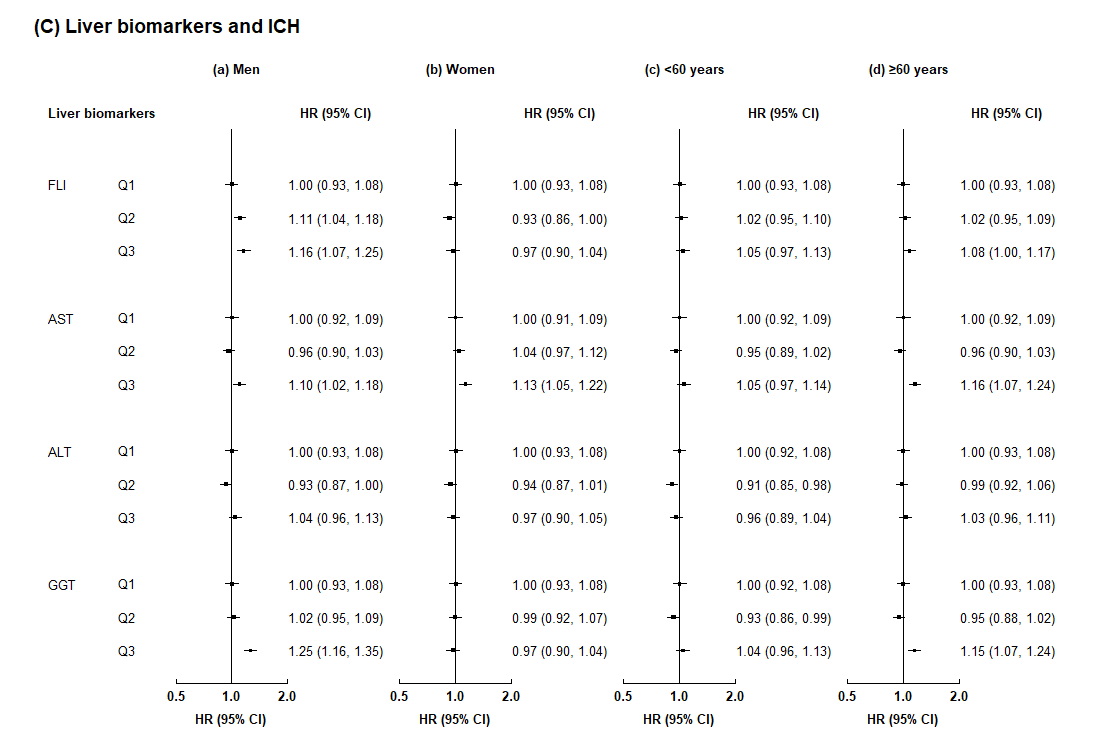
**

# Supplementary Table 1. Associations of liver biomarkers with risk of CVD

|  | **HR (95% CI)** | | |
| --- | --- | --- | --- |
| Liver biomarkers | **MI** | **IS** | **ICH** |
| **FLI per 1-SD** | 1.17 (1.13, 1.21) | 1.16 (1.13, 1.19) | 1.04 (1.01, 1.08) |
| **AST per 1-SD** | 1.08 (1.05, 1.12) | 0.93 (0.90, 0.97) | 1.11 (1.08, 1.13) |
| **ALT per 1-SD** | 1.07 (1.04, 1.11) | 0.98 (0.95, 1.01) | 1.04 (1.01, 1.07) |
| **GGT per 1-SD** | 1.08 (1.05, 1.11) | 1.02 (0.99, 1.05) | 1.10 (1.08, 1.12) |

Abbreviations: MI=myocardial infarction, IS=ischemic stroke, ICH=intracerebral hemorrhage, FLI=fatty liver index, AST= aspartate aminotransferase, ALT= alanine aminotransferase, γ-glutamyltransferase

Cox proportional hazards regression models were used in the analysis. The model is adjusted for age, sex, regions, education, smoking, and alcohol.

# Supplementary Table 2. Associations of individual high-risk lifestyle risk factors with risk of CVD

|  | **HR (95% CI)** | | |
| --- | --- | --- | --- |
| High-risk lifestyle factors | **MI** | **IS** | **ICH** |
| **Smoking** | 1.27 (1.15, 1.41) | 1.07 (0.99, 1.17) | 0.95 (0.88, 1.04) |
| **Alcohol** | 1.12 (1.04, 1.21) | 1.13 (1.07, 1.20) | 1.18 (1.10, 1.26) |
| **Physical inactivity** | 1.05 (0.95, 1.15) | 1.05 (0.98, 1.13) | 1.08 (1.00, 1.17) |
| **Central adiposity** | 1.38 (1.28, 1.48) | 1.34 (1.27, 1.42) | 1.08 (1.01, 1.16) |

Cox proportional hazards regression models were used in the analysis. The model is adjusted for age, sex, regions, education, and the other three high-risk lifestyle factors.

# Supplementary Table 3. Associations of liver biomarkers with risk of CVD

|  | **HR (95% CI)** | | | | | | |
| --- | --- | --- | --- | --- | --- | --- | --- |
| Liver biomarkers | **Overall** |  | **rs738409** | |  | **High-risk lifestyle factors** | |
|  |  |  | **0 alleles** | **1-2 alleles** |  | **0-1 factors** | **≥2 factors** |
| **MI** | | | | | | | |
| **NAFLD** | 1.43 (1.30, 1.57) |  | 1.35 (1.17, 1.56) | 1.48 (1.30, 1.68) |  | 1.06 (0.69, 1.63) | 1.39 (1.26, 1.54) |
| *p-interaction* |  |  | *0.29* | |  | *0.28* | |
| **MAFLD** | 1.42 (1.30, 1.55) |  | 1.33 (1.16, 1.52) | 1.48 (1.31, 1.66) |  | 1.12 (0.75, 1.67) | 1.38 (1.26, 1.52) |
| *p-interaction* |  |  | *0.21* | |  | *0.35* | |
| **AST** | 1.13 (1.05, 1.21) |  | 1.24 (1.10, 1.39) | 1.06 (0.96, 1.17) |  | 1.09 (0.92, 1.30) | 1.14 (1.05, 1.24) |
| *p-interaction* |  |  | *0.11* | |  | *0.68* | |
| **ALT** | 1.12 (1.04, 1.21) |  | 1.20 (1.07, 1.34) | 1.07 (0.97, 1.18) |  | 1.04 (0.88, 1.24) | 1.12 (1.03, 1.21) |
| *p-interaction* |  |  | *0.13* | |  | *0.79* | |
| **GGT** | 1.27 (1.18, 1.37) |  | 1.32 (1.18, 1.48) | 1.24 (1.13, 1.37) |  | 1.22 (1.02, 1.45) | 1.26 (1.16, 1.37) |
| *p-interaction* |  |  | *0.50* | |  | *0.82* | |
| **IS** | | | | | | | |
| **NAFLD** | 1.25 (1.16, 1.35) |  | 1.24 (0.81, 1.39) | 1.25 (1.13, 1.39) |  | **1.57 (1.21, 2.03)** | **1.16 (1.07, 1.26)** |
| *p-interaction* |  |  | *0.77* | |  | ***0.03*** | |
| **MAFLD** | 1.24 (1.15, 1.33) |  | 1.22 (1.10, 1.35) | 1.25 (1.14, 1.37) |  | **1.53 (1.20, 1.96)** | **1.16 (1.07, 1.24)** |
| *p-interaction* |  |  | *0.75* | |  | ***0.02*** | |
| **AST** | 0.93 (0.88, 0.98) |  | 0.99 (0.90, 1.08) | 0.89 (0.82, 0.96) |  | 0.92 (0.81, 1.04) | 0.93 (0.87, 0.99) |
| *p-interaction* |  |  | *0.05* | |  | *0.74* | |
| **ALT** | 1.03 (0.97, 1.09) |  | 1.04 (0.95, 1.14) | 1.03 (0.95, 1.10) |  | 0.98 (0.87, 1.10) | 1.03 (0.96, 1.09) |
| *p-interaction* |  |  | *0.78* | |  | *0.21* | |

|  |  |  |  | |  |  | |
| --- | --- | --- | --- | --- | --- | --- | --- |
|  | **HR (95% CI)** | | | | | | |
| Liver biomarkers | **Overall** |  | **rs738409** | |  | **High-risk lifestyle factors** | |
|  |  |  | **0 alleles** | **1-2 alleles** |  | **0-1 factors** | **≥2 factors** |
| **GGT** | 1.18 (1.11, 1.25) |  | 1.14 (1.04, 1.25) | 1.21 (1.12, 1.30) |  | 1.19 (1.05, 1.34) | 1.14 (1.07, 1.22) |
| *p-interaction* |  |  | *0.56* | |  | *0.66* | |
| **ICH** | | | | | | | |
| **NAFLD** | 1.12 (1.02, 1.23) |  | **0.96 (0.82, 1.12)** | **1.24 (1.10, 1.39)** |  | **1.38 (0.98, 1.95)** | **1.08 (0.98, 1.19)** |
| *p-interaction* |  |  | ***0.01*** | |  | ***0.04*** | |
| **MAFLD** | 1.12 (1.03, 1.22) |  | **0.98 (0.85, 1.12)** | **1.22 (1.10, 1.36)** |  | **1.38 (0.99, 1.91)** | **1.08 (0.99, 1.18)** |
| *p-interaction* |  |  | ***0.01*** | |  | ***0.04*** | |
| **AST** | 1.02 (0.96, 1.09) |  | 1.05 (0.95, 1.16) | 1.00 (0.92, 1.09) |  | 1.03 (0.89, 1.19) | 1.01 (0.95, 1.09) |
| *p-interaction* |  |  | *0.54* | |  | *0.45* | |
| **ALT** | 0.98 (0.92, 1.05) |  | 0.95 (0.86, 1.05) | 1.00 (0.93, 1.09) |  | 0.98 (0.85, 1.14) | 0.97 (0.91, 1.04) |
| *p-interaction* |  |  | *0.33* | |  | *0.61* | |
| **GGT** | 1.06 (0.99, 1.12) |  | 1.02 (0.93, 1.13) | 1.08 (0.99, 1.16) |  | 1.11 (0.96, 1.28) | 1.02 (0.95, 1.10) |
| *p-interaction* |  |  | *0.29* | |  | *0.19* | |

Cox proportional hazards regression models were used in the analysis. The model is adjusted for age, sex, regions, education, smoking, and alcohol.

# Supplementary Table 4. *P*-values for interaction by sex and age

|  | **FLI** | **AST** | **ALT** | **GGT** |
| --- | --- | --- | --- | --- |
| *Interaction by sex* | |  |  |  |
| MI | 0.40 | 0.49 | 0.87 | 0.45 |
| IS | 0.48 | 0.66 | 0.35 | 0.11 |
| ICH | **0.02** | 0.74 | 0.40 | **<0.01** |
| *Interaction by age* | |  |  |  |
| MI | 0.15 | 0.10 | **0.01** | **<0.01** |
| IS | 0.72 | 0.56 | 0.06 | 0.51 |
| ICH | 0.13 | 0.20 | 0.25 | 1.00 |

# Supplementary Table 5. Associations of liver biomarkers with carotid plaque

|  | **OR (95% CI)** | | | | | | |
| --- | --- | --- | --- | --- | --- | --- | --- |
| Liver | **Overall** |  | **rs738409** | |  | **High-risk lifestyle factors** | |
| biomarkers |  |  | **0 alleles** | **1-2 alleles** |  | **0-2 factors** | **≥3 factors** |
| **NAFLD** | 2.36 (1.12, 4.96) |  | 1.20 (0.27, 5.27) | 4.16 (1.40, 12.38) |  | 2.29 (0.79, 6.66) | 2.33 (0.58, 9.40) |
| *p-interaction* |  |  | *0.18* | |  | *0.90* | |
| **MAFLD** | 2.36 (1.12, 4.96) |  | 1.20 (0.27, 5.27) | 4.16 (1.40, 12.38) |  | 2.29 (0.79, 6.66) | 2.33 (0.58, 9.40) |
| *p-interaction* |  |  | *0.18* | |  | *0.90* | |
| **AST** | 0.67 (0.38, 1.19) |  | 0.28 (0.10, 0.80) | 1.25 (0.54, 2.90) |  | 0.66 (0.31, 1.41) | 0.76 (0.25, 2.25) |
| *p-interaction* |  |  | *0.09* | |  | *0.36* | |
| **ALT** | 0.96 (0.55, 1.66) |  | 0.41 (0.16, 1.07) | 1.27 (0.56, 2.90) |  | 0.81 (0.40, 1.66) | 0.97 (0.33, 2.79) |
| *p-interaction* |  |  | *0.15* | |  | *0.24* | |
| **GGT** | 1.61 (0.91, 2.86) |  | 1.78 (0.67, 4.69) | 2.00 (0.84, 4.78) |  | 1.83 (0.84, 3.98) | 1.23 (0.41, 3.65) |
| *p-interaction* |  |  | *0.50* | |  | *0.74* | |

Logistic regression models were used in the analysis. The model is adjusted for age, sex, regions, education, smoking, and alcohol.

High-risk lifestyle factors are classified into two groups (0-2 and 3+ factors) because of the small number of participants with data of subclinical atherosclerosis.
